# Advances in verbal autopsy: pragmatic optimism or optimistic theory?

**DOI:** 10.1186/1478-7954-9-24

**Published:** 2011-08-01

**Authors:** Edward Fottrell

**Affiliations:** 1 Umeå Centre for Global Health Research, Department of Public Health and Clinical Medicine, Umeå University, Sweden; 2 Centre for International Health and Development, Institute of Child Health, University College London, UK

## Commentary

In recent decades, verbal autopsy (VA) methods have been increasingly used to identify likely causes of death in settings where the majority of deaths occur without medical attention or certification as to cause [1]. Developments in the 1980s and 1990s advanced the conceptual and methodological aspects of the science considerably but fell short of providing a clear message about best practices for those who rely on VA data [2-10]. There has been a hiatus of methodological development since then, due in part to persistent, narrow assumptions as to the desire and need for cause of death data and unrealistic evaluation standards. This has not limited the application of VA methods in the world's poorest settings, but it has almost certainly limited the usefulness and comparability of the data. There remains scarce evidence on which to base choice of methods at the various stages of the VA data process. However, the first ever Global Congress on Verbal Autopsy, held in Bali, Indonesia in February 2011, represents a resurgence of methodological and conceptual developments - VA is arguably one of the most important fields in global health today.

Methodological development in recent years, particularly in relation to probabilistic interpretation of VA data, has brought VA into an exciting era that is creating new opportunities for reliable, timely, and useful cause-specific mortality measurement. A shift away from limited individual-level and clinical paradigms towards population-based epidemiological thinking and public health utility has been characterized by a flurry of new methodological thinking and innovations from relatively small groups of researchers. Among these like-minded researchers, however, there is risk of a divide between pragmatic optimists and optimistic theorists. The pragmatic optimists are driven by the realities, perils, and pitfalls of real-life health measurement in low-income settings and strive to enhance health knowledge with methods that are good enough to fill data gaps reliably and efficiently. The optimistic theorists, whose methodological developments are often theoretically superior, are often far from offering practical solutions to those on the ground who need to know the major burdens of cause-specific mortality in their populations simply, quickly, and cheaply in the absence of pre-existing data and where "true validity" is difficult to establish. Such dichotomization is perhaps somewhat artificial, but there is a real risk that unrealistic standards and expectations in method development and evaluation will become the enemy of good enough methods that are able to provide essential data to those who need it.

The Global Congress on VA was attended by over 100 delegates representing numerous agencies and academic institutions from around the globe. This level of participation illustrates not only the persistent desire to know who died from what in the world's poorest settings, but also a desire for clear leadership on what the best methods are and how to use them. Whilst methodological favoritism, ideology, and competition can be a threat to an objective answer to this question, such factors are, in reality, likely to be minor and secondary to the more fundamental difficulty of recognizing the range of users who need cause of death data and what data they need.

Differing cause of death data needs have been well described previously and the nonexistence of a one-size-fits-all solution to all needs is likely to persist [11]. This statement is grounded in recognition of the realities of health metrics in the world's poorest populations and the imperfect world of lay-reported signs and symptoms, dubious record keeping, and biases in remembering, reporting, and recording certain events. It is in recognition of these realities that the gap between the pragmatic optimists and the optimistic theorists may grow: the first believing that method development cannot necessarily rely on pre-existing data and should be evaluated in terms of comparability to reference (but not gold) standards, plausibility in relation to given knowledge, and the ability to characterize population-level cause-specific mortality well enough for the use of such data for planning, monitoring, and evaluation; the latter continuing on a quest for true validity, believing that this does exist in relation to cause of death.

Validation is of course desirable for any method, but do global cause of death gold standards for the validation of VA methods really exist? Hospital-based data have been used in the past and continue to be used for validation studies; however, deaths for which such data are available are not representative of deaths in the majority of individuals who live their lives, get sick, and die with limited or no contact with formal health services. Similarly, the symptom profiles of individuals who die in the community in the absence of health care, or at least the symptoms that are recalled and reported by relatives of those individuals during VA interviews, are likely to be considerably different from those who were informed by contact with medical services. Even if sophisticated methods attempt to adjust for this, the costs of this alchemy are high and the gold is without doubt alloyed, limiting its relevance for true community-based populations and those who must plan services for such populations. This fact does not mean that such comparisons are not valuable to a certain extent - they may highlight gross inconsistencies, for example.

A key achievement of the Bali congress was a "Bali Declaration" that physician review of VA data as the default method of choice for all VA interpretation should be a thing of the past. On this, most experts agreed, and this declaration represents a substantial step forward, which will have untold implications for the timeliness and utility of VA-derived cause of death data. Such unity in communication on best practice in VA methods from the world's leaders in the field is timely and commendable. The same unified voice on the best method for VA interpretation that many might have hoped to hear in Bali is not yet loud and clear, and indeed better methods are likely to evolve over the coming years. Before this can happen, however, there needs to be agreement on flexibility in evaluation standards, recognizing that the utility of a method is highly dependent on who wants the data and what they want to do with them. Single-mindedness with regards to absolute measures of validity, true gold standards, and the utility of data are likely to hinder ongoing developments in VA at the expense of immediate public health benefits and to the detriment of conceptual advances that have been made in recent years.
